# The Diagnostic Value of the Combined 3D Pseudo-Continuous Arterial Spin Labeling and Diffusion Kurtosis Imaging in Patients With Binswanger’s Disease

**DOI:** 10.3389/fnins.2022.853422

**Published:** 2022-06-30

**Authors:** Xiaoyi He, Weiqiang Dou, Hao Shi

**Affiliations:** ^1^Department of Radiology, The First Affiliated Hospital of Shandong First Medical University and Shandong Provincial Qianfoshan Hospital, Jinan, China; ^2^Department of Radiology, The Second Affiliated Hospital of Shandong University of Traditional Chinese Medicine, Jinan, China; ^3^MR Research, GE Healthcare, Beijing, China

**Keywords:** Binswanger’s disease, subcortical arteriosclerotic encephalopathy, diffusion kurtosis imaging, arterial spin labeling, magnetic resonance (MR)

## Abstract

**Background and Purpose:**

The clinical diagnosis of Binswanger’s disease (BD), a chronic progressive form of subcortical vascular dementia, remains challenging. 3D pseudo-continuous arterial-spin-labeling (pcASL) and diffusion kurtosis imaging (DKI) can quantitatively reveal the microcirculation changes and heterogeneity of white matter (WM), respectively. We thus aimed to determine the diagnostic value of the combined 3D-pcASL and DKI in BD.

**Materials and Methods:**

A total of 35 patients with BD and 33 healthy controls underwent 3D-ASL and DKI experiments. The perfusion parameter of cerebral blood flow (CBF), diffusion parameters of fractional anisotropy (FA), mean/axial/radial diffusivity (MD/Da/Dr), and kurtosis parameters of anisotropy fraction of kurtosis (FAk) and mean/axial/radial kurtosis MK/Ka/Kr were obtained to quantitatively measure the parametric distributions of functional brain subregions. One-way analysis of variance and *post hoc t*-test were applied to explore the different distributions of DKI/ASL-derived parameters among brain subregions of BD. In addition, all region-specific DKI/ASL parameters were separately analyzed in Pearson correlation analysis to investigate the relationship with Mini-Mental State Examination (MMSE), a typical clinical scale for cognitive function assessment in patients with BD.

**Results:**

FA/FAk/MK/Ka/Kr was significantly declined in all WM hyperintensities (WMHs) of BD compared with healthy controls, while the corresponding MD/Da/Dr was significantly increased (all *p* < 0.005). In addition, significant changes, similar to the WMHs of patients with BD, were also observed in almost all DKI parameters in WM normal areas and genu/splenium of the corpus callosum (GCC/SCC) in BD (*p* < 0.005). Finally, CBF was significantly reduced in all of the above regions we measured in patients with BD (*p* < 0.005). For patients with BD, MMSE showed a negative correlation with MD/Da in thalamus (*r* = −0.42/−0.58; *p* < 0.05), and a positive correlation with CBF in PWM/TWM (*r* = 0.49/0.39; *p* < 0.05). Using receiver operating characteristic (ROC) analysis, FA/FAk/Kr in GCC, CBF/FA/Dr/FAk in SCC, MD/Da/Ka in thalamus, and the combined FA/MD/Dr/CBF in TWM showed high accuracy [area under the curves (AUCs) 0.957/0.946/0.942/0.986] in distinguishing patients with BD from healthy controls.

**Conclusion:**

We found that combined DKI and 3D-ASL are helpful in diagnosing patients with BD, especially with FA, MD, Dr, and CBF in the temporal WM region. Additionally, the kurtosis parameters of DKI can sensitively monitor the potentially damaged WM areas in patients with BD patients, adding complementary clinical value.

## Introduction

Binswanger’s disease (BD) is a chronic progressive form of subcortical vascular dementia (SIVD). In recent years, as the incidence of BD has dramatically increased, clear pathological measurement is increasingly considered essential in diagnosing BD (Caplan and Schoene, 1978). But, the intrinsic properties, such as invasiveness, limit its wide application in clinics.

As an alternative, non-invasive magnetic resonance imaging (MRI), such as fluid-attenuated inversion recovery (FLAIR), has also been reported to visualize multiple lacunar infarctions and diffused white matter hyperintensities (WMHs) around the bilateral ventricular in patients with BD (Akiguchi et al., 2014). However, with the anatomic MR images, the diagnosis of BD remains clinically challenging and is usually misdiagnosed as leukoaraiosis (LA; Huisa and Rosenberg, 2014) due to similar imaging features. To address this issue, a more effective MRI method is desired since the progressive state of disease reflected has a positive effect on follow-up treatment for patients with BD (Rosenberg et al., 2015).

Although diffusion tensor imaging (DTI) can provide diffusivity characteristics of white matter (WM) nerve fibers, the heterogeneity of water molecules is difficult to explore (Tuch et al., 2002). Diffusion kurtosis imaging (DKI), as an extended diffusion imaging technique, employs multiple *b* values and diffusion directions to quantify the non-Gaussian distribution of water diffusion (Jensen and Helpern, 2010). In addition to DTI-relevant parameters, DKI can provide kurtosis-relevant parameters, including mean kurtosis (MK), anisotropy fraction of kurtosis (FAk), axial kurtosis (Ka), and radial kurtosis (Kr), which can sensitively reflect the non-Gaussian diffusion characteristics of water molecules in WM lesions. Also, DKI parameters have higher sensitivity and stability in monitoring local microstructures in the brain compared with DTI parameters, which is of great significance for monitoring the microstructural changes of WM diseases (Zhong et al., 2017). Previous studies have shown that DKI can efficiently detect WM alterations in diseases such as chronic brain stroke, demyelination, and spontaneous remyelination (Umesh Rudrapatna et al., 2014; Zhu et al., 2015; Guglielmetti et al., 2016; Yang et al., 2021).

Other functional MRI techniques such as magnetic resonance spectroscopy (MRS) and dynamic contrast-enhanced MRI (DCE-MRI) have been applied to explore the pathophysiological mechanism of BD (Huisa et al., 2015; Barry Erhardt et al., 2019; Fu et al., 2020; Xin et al., 2020). To a certain extent, these techniques are able to measure WM damages and blood-brain barrier destructions in patients with BD. However, not high stability of MRS and contrast agent required in DCE-MRI limit their clinical applications. In comparison, 3D pseudo-continuous arterial spin labeling (3D-pcASL), as a contrast-free perfusion technique, uses magnetically labeled endogenous arterial blood as a tracer to quantify cerebral blood flow (CBF) of microscopic structures. The quantitative CBF has been reported to allow accurate evaluation of cerebral perfusion in elderly patients with LA (Schuff et al., 2009; Yang et al., 2021).

Based on these advantages of DKI and ASL, we assumed that DKI can potentially explore the heterogeneity of WM fibers in patients with BD, and 3D-ASL can quantitatively analyze the changes of microcirculation in functional brain subregions for patients with BD. Few studies may have applied both techniques in BD diagnosis so far.

Therefore, this study aimed to investigate whether combined DKI and ASL can reveal pathophysiological changes and further diagnose patients with BD. The distribution characteristics of water molecule diffusion and the changes in blood perfusion were systematically explored in functional subregions closely related to the symptoms of patients with BD, including frontal and parietal WM for thinking and language ability, basal ganglia for motor behavioral functions, thalamus for cognitive functions, temporal WM and hippocampus for memory, and sensory language functions in patients with BD, and compared with those in healthy controls.

## Materials and Methods

### Subjects

This study has been approved by the Ethics Committee of The First Affiliated Hospital of Shandong First Medical University (Shandong Qianfoshan Hospital) [2021] Lun Shen Zi (S068). All subjects have signed an informed consent form. Between October 2019 and August 2020, 35 consecutive patients with BD (19 men, 72.2 ± 6.6 years) were recruited from Shandong Qianfoshan Hospital. Moreover, 33 age and gender-matched healthy subjects (15 men, 70.6 ± 6.8 years) were enrolled as healthy controls (Table 1). Mini-Mental State Examination (MMSE) score was used to assess the cognitive function of patients with BD and healthy controls (Table 1).

**TABLE 1:** Clinical information about comparison of age and gender between the patients with Binswanger’s disease (BD) and healthy controls using *t*-test and chi-square test, respectively.

Groups	*n*	Age (years)	Gender (men/women)	Years of education	MMSE score
BD	35	(72 ± 6.59)	19/16	2∼15 (9.5 ± 0.32)	13∼26 (19.54 ± 3.76)
Healthy controls	33	(70 ± 6.78)	15/18	4∼16 (9.6 ± 0.57)	27∼30 (28.53 ± 0.89)
Statistical analysis		*t* = 0.9819 *P* = 0.3297	χ^2^ = 0.529 *P* = 0.467		

All patients, who met the Caplan criteria (Caplan and Gomes, 2010) for BD, were diagnosed by experienced radiologists and underwent clinical examinations. The Caplan criteria mainly include (1) previous hypertension and other factors that promote vascular diseases in middle-aged and elderly people; (2) progressive cognitive impairment; (3) the cumulative presence of localized neurological symptoms; (4) acute strokes history; (5) presence of extensive WM damages (including subcortical deep WM of peripheral ventricles, radiating crown, and centrum semiovale) in MRI, especially multiple WMHs in T2-FLAIR imaging; (6) severe atrophy of WM around bilateral ventricles; and (7) multiple lacunar infarcts. The exclusion criteria were defined as follows: (1) existence of disability owing to neurological disorders other than BD, such as Alzheimer’s disease, Pick disease, psychosis, multiple sclerosis, or progressive multifocal leucoencephalopathy; (2) other types of specific leukoencephalopathy; and (3) contraindications for MRI or refused to undergo MRI measurements.

### Magnetic Resonance Imaging Acquisition

All MRI examinations were performed at 3.0 Tesla (DISCOVERY MR750w, GE Healthcare, Shanghai, China, United States) using an 8-channel head phased array coil. High-resolution T1-weighted anatomical imaging (BRAVO), T2-FLAIR, DKI, and 3D-pcASL imaging were performed on each participant.

A single-shot spin-echo echo-planar imaging (SE-EPI)-based DKI sequence was implemented in the axial plane to measure the whole brain in a supine position. The correspondingly applied scan parameters were of repetition time (TR) = 2,000 ms, echo time (TE) = 72.20 ms, flip angle (FA) = 90°, matrix = 256 × 256; slice thickness = 5 mm, slice spacing = 1.5 mm, and field of view (FOV) = 260 mm × 260 mm. In addition, five *b* values (400, 800, 1,200, 1,600, and 2,000 s/mm^2^), 15 directions at each *b* value, as well as a *b* of 0 s/mm^2^ were used. The scan time was 7 min 30 s.

A spiral fast spin-echo-based 3D-pcASL sequence was acquired for brain perfusion measurement. The correspondingly applied scan parameters were of TR/TE, 4,632 ms/10.54 ms; matrix = 128 × 128; slice thickness = 4 mm; FOV = 240 mm × 240 mm; labeling duration, 1,450 ms, and post-labeling delay time, 1,525 ms; NEX = 3.00; and effective resolution = 3.64 mm × 3.64 mm. The scan time was 4 min 29 s.

### Image Analysis

All acquired DKI and 3D-pcASL images were analyzed in a GE advanced workstation using the vendor-provided DKI and ASL postprocessing software (GE functool 4.6 software, United States) (Jensen and Helpern, 2010; Alsop et al., 2015). Multiple DKI-derived parametric mapping, i.e., fractional anisotropy (FA), diffusion-related parameters of mean/axial/radial diffusivity (MD/Da/Dr), kurtosis-related parameters of FAk, Mk, Ka, and Kr maps, and the CBF mapping from 3D-pcASL imaging were obtained, respectively.

Three well-trained radiologists (AA, 30 years of seniority; BB, 25 years of seniority; CC, 14 years of seniority) were employed independently to assess the anatomic and functional MR images. Based on BRAVO T1w images and DKI maps at *b* = 0 s/mm^2^, main neuropathological diseases, such as large-vessel infarctions and tumors, were first avoided in the whole brain. Multiple regions of interest (ROIs) for WM hyperintensities of patients with BD, WM normal areas of patients with BD, and healthy controls were then manually drawn on functional brain subregions and then copied on the ASL/DKI-derived parametric maps. Average values of each parameter per ROI over three measures were further used in the statistical analysis.

**FIGURE 1:**
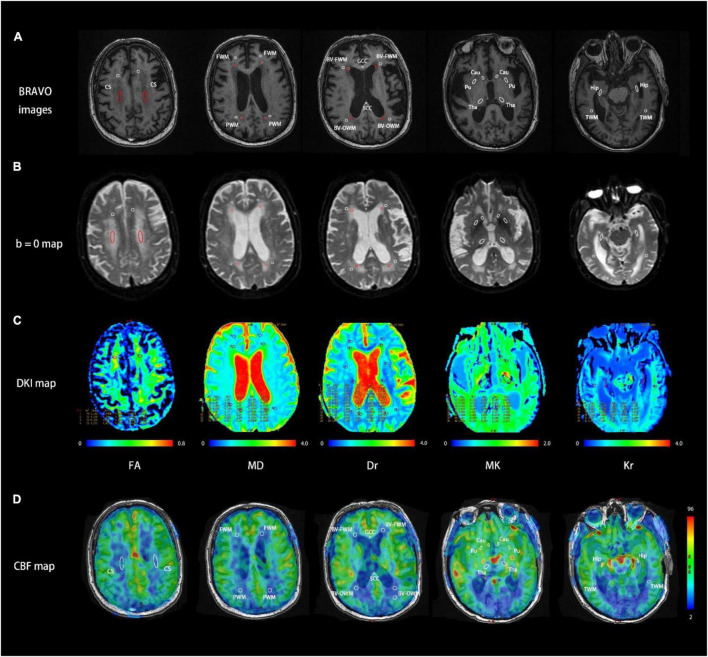
Representative MR images at five consecutive slices of a patient with Binswanger’s disease (BD) acquired using BRAVO image **(A)**, DKI *b* = 0 map **(B)**, DKI parametric maps **(C)**, and cerebral blood flow (CBF) map **(D)**. Region of interest (ROI) settings. CS, centrum semiovale; GCC/SCC, genu/splenium of the corpus callosum; FWM/PWM/TWM, frontal/parietal/temporal white matter; BV-FWM/BV-OWM, frontal/occipital white matter around lateral ventricle; Cau, caudate nucleus; Pu, putamen; Tha, thalamus; Hip, hippocampus. Each ROI was also denoted with BRAVO images, *b* = 0 maps, and DKI/ASL-derived parameters maps. The red ROIs in *b* = 0 maps and BRAVO images represent white matter hyperintensities and the white ROIs in both maps represent normal areas in MRI.

With reference to bilateral ventricles, anterior commissure, and hippocampal body (Wei et al., 2016), all ROIs were selected in circular or ovoid shape based on T1w images (BRAVO) and DKI maps at *b* = 0 s/mm^2^. Referring to the normal areas and lesion areas (low signal areas) on T1w images, the corresponding ROIs were drawn on b0 images and copied onto the corresponding parametric maps of DKI. For the BD group, we selected 32 ROIs per subject, including 10 ROIs for lesion areas and 22 ROIs for normal areas. For the healthy control group, 22 ROIs for normal areas were chosen per subject. The matched ROIs between patients with BD and healthy controls were comparable in location and size. Detailed geometric information of each ROI and selection scheme are shown in Figure 1 and Table 2.

**TABLE 2:** Geometric information of each region of interest (ROI) selected in multiple brain functional subregions.

Region (mm^2^)	Centrum semiovale	GCC/SCC/TWM	FWM/PWM/BV-FWM/BV-OWM	Caudate nucleus	Putamen	Thalamus	Hippocampus
**Groups**							
Lesion	60–80	25–30	25–30				
Normal region	25–30		25–30	30	40	70	50

*GCC/SCC, genu/splenium of the corpus callosum; FWM/PWM/TWM, frontal/parietal/temporal WM; BV-FWM/BV-OWM, frontal/occipital WM around bilateral ventricle. The numbers in the table represent the size of ROI with the unit of mm^2^.*

### Statistical Analysis

All statistical analyses were performed using the IBM SPSS 22.0 statistical software and the GraphPad Prism 8.0.2 software. The demographic and clinical parameters were tested for normality utilizing the Kolmogorov–Smirnov test and Shapiro–Wilk test. The Student’s *t-*test and chi-square test were used to assess differences in age and gender between patients with BD and healthy controls, respectively. The intra-class correction coefficient (ICC) analysis was used to evaluate the interobserver agreement of ROI-specific DKI/ASL parameter measurement over three experts. ICC > 0.75 was considered good reproducibility. One-way analysis of variance (one-way ANOVA) followed by the *post hoc t*-test analysis was used to assess differences of FA, MD, Da, Dr, FAk, Mk, Ka, and Kr in ROIs among three groups (WM hyperintensities of patients with BD, WM normal areas of patients with BD, and healthy controls). Kruskal–Wallis test and Dunn test were used to analyze the data without normal distribution and review every single variance. *p*-value < 0.005 was considered the significant different values after multiple group Bonferroni correction. Additionally, comparisons of DKI/ASL parameters were performed using a paired-sample *t-*test between the left and right hemispheric ROIs for patients with BD or for healthy controls and using a single-sample *t-*test between WM normal areas of patients with BD and healthy controls. The latter without normal distribution was tested utilizing the Mann–Whitney test. Binary logistic regression analysis was used in the receiver operating characteristic (ROC) analysis with combined DKI/ASL parameters. Pearson’s correlation was used to assess the respective relationship between each DKI/ASL parameter and MMSE score in patients with BD. The MMSE, a typical clinical scale for cognitive function assessment, includes the following 7 aspects: time orientation, place orientation, immediate memory, attention and calculation, delayed memory, language, and visual space. ROC analysis was conducted to determine the efficacy of DKI/ASL parameters in distinguishing patients with BD from healthy controls. A *p*-value < 0.05 was considered as the threshold of statistical significance.

## Results

### Interobserver Agreement Analysis

Excellent interobserver agreement over three experts was confirmed by high ICC values obtained for each measured DKI parameter, including FA (0.895), MD (0.872), Da (0.839), Dr (0.887), FAk (0.745), MK (0.860), Ka (0.768), and Kr (0.861). Meanwhile, a similar result was also revealed for 3D ASL-derived CBF with a high ICC of 0.936.

### Comparison of Diffusion Kurtosis Imaging/Arterial-Spin-Labeling Parameters Among White Matter Hyperintensities of Patients With Binswanger’s Disease, White Matter Normal Areas of Patients With Binswanger’s Disease, and Healthy Control Groups

**FIGURE 2:**
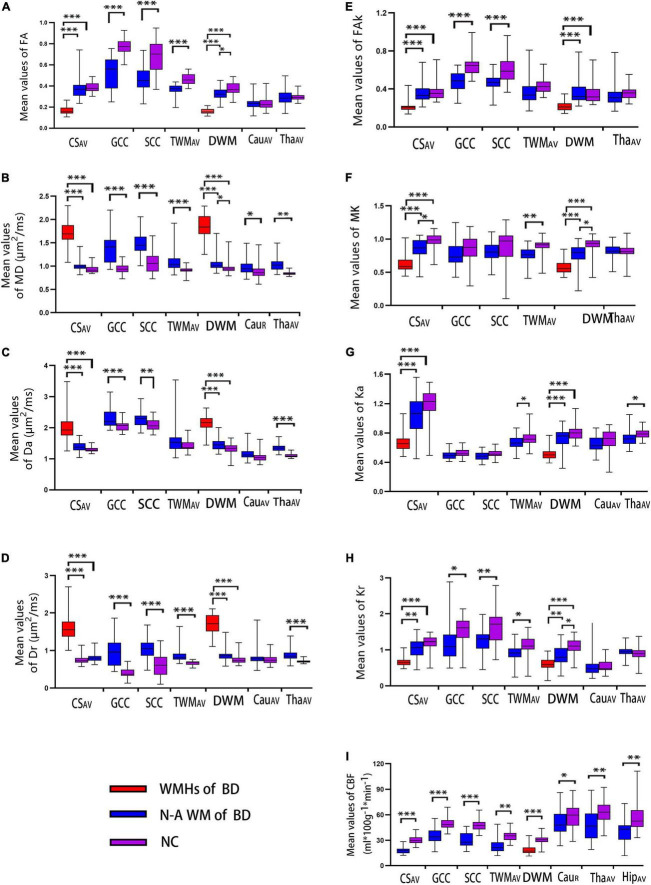
Box and whiskers graphs of DKI measurements (**A–I** represent the FA, MD, Da, Dr, FAk, MK, Ka, and Kr, respectively.) among the three groups (WMHs of BD, WM normal-areas of BD, and NC) and ASL measurement (CBF) between BD and NC groups over different subregions of the brain. The solid line inside the box represents the median value, whereas the edges represent the 25th and 75th percentiles. A straight line (bar) on each box indicates the range of data distribution. BD, Binswanger’s disease; WMHs of BD, white matter hyperintensities of patients with BD; N-A WM of BD, white matter normal areas of patients with BD; NC, healthy control; R, the right-hemispheric side; AV, average of bilateral ROI measurements; CS, centrum semiovale; GCC/SCC, genu/splenium of the corpus callosum; DWM, deep white matter, including the frontal/parietal white matter and the WM around bilateral ventricle (in the figure, bilateral ventricle around frontal WM is used as the representative area of DWM); TWM, temporal white matter; Cau, caudate nucleus; Tha, thalamus; Hip, hippocampus. Significant difference: *p < 0.005; ^**^p < 0.001; ^***^p < 0.0001.

As shown in Figure 2, FA/FAk/MK/Ka/Kr was significantly declined in all WM hyperintensities (WMHs) of BD compared with healthy controls, while the corresponding MD/Da/Dr were significantly increased (all *p* < 0.005). In addition, significant changes, similar to the WMHs of patients with BD, were also observed in FA/FAk/Kr and all diffusion parameters of GCC and SCC, FA/MD/Dr/MK/Ka/Kr of TWM, MD of right Cau, as well as MD/Da/Dr/Ka of the thalamus in patients with BD, relative to healthy controls (all *p* < 0.005). Finally, CBF was significantly reduced in all of the above regions we measured for patients with BD, relative to healthy controls (*p* < 0.005). More detailed results are presented in Supplementary Figure 2.

### Correlation Analyses Among Diffusion Kurtosis Imaging- and Arterial-Spin-Labeling-Derived Parameters and Mini-Mental State Examination Scores of Patients With Binswanger’s Disease

**FIGURE 3:**
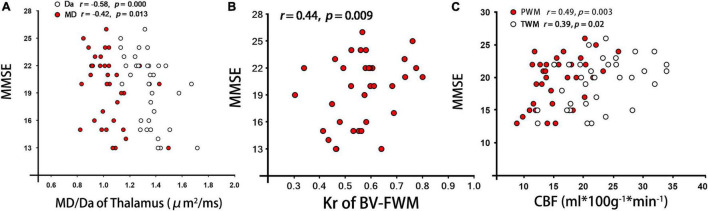
Correlations analyses between each DKI/ASL derived parameter and MMSE scores from patients with BD. **(A–C)** represent the correlation of MMSE with MD/Da of Thalamus, Kr of BV-FWM, and CBF of PWM/TWM, respectively. Note: PWM/TWM, parietal/temporal white matter; MMSE, Mini-Mental State Examination.

As shown in Table 3 and Figure 3, for patients with BD, the CBF values in WM regions with hyperintensities around bilateral ventricle showed a significant correlation with MK and Kr (BV-FWM, *r* = 0.50/0.51, *p* = 0.002/0.001; BV-OWM, *r* = 0.47/0.52, *p* = 0.004/0.002). Moreover, the CBF values in thalamus, as WM normal areas, were also significantly correlated with MD, Da, and Dr (*r* = −0.59/−0.61/−0.55, *p* < 0.001). In addition, for patients with BD, MMSE scores were negatively correlated with MD/Da of thalamus (*r* = −0.42/−0.58, *p* = 0.013/0.000, Figure 3A), and positively correlated with FA/Kr of frontal WM around bilateral ventricle (*r* = 0.48/0.44, *p* = 0.003/0.009, Figure 3B) and with CBF of parietal/temporal WM (*r* = 0.49/0.39, *p* = 0.003/0.02, Figure 3C).

**TABLE 3:** Correlations among DKI-derived parameters, ASL-derived parameters, and MMSE score obtained from patients with BD.

Groups	CBF value of BV-FWM and the value of MK/Kr	CBF value of BV-OWM and the value of MK/Kr	MD/Da/Dr value and CBF of thalamus	MD/Da value in thalamus and MMSE score of BD	Kr value in BV-FWM and MMSE score of BD	CBF value in PWM/TWM and MMSE score of BD
*r* value	0.50/0.51	0.47/0.52	−0.59/−0.61/−0.55	−0.58/−0.42	0.44	0.49/0.39
*P* value	0.002/0.001	0.004/0.002	0.000	0.000/0.013	0.009	0.003/0.020

*PWM/TWM, parietal/temporal WM; BV-FWM/BV-OWM, lateral ventricle around frontal and occipital WM; MMSE, Mini-Mental State Examination.*

### Receiver Operating Characteristic Analyses of Diffusion Kurtosis Imaging/Arterial-Spin-Labeling Parameters in the Diagnosis of Binswanger’s Disease

**FIGURE 4:**
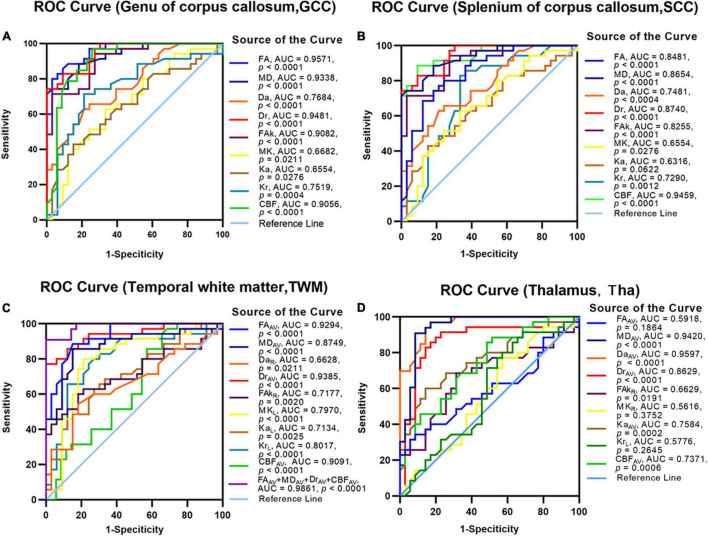
The receiver operating characteristic (ROC) analysis to assess the efficacy of DKI/ASL-derived parameters for discriminating patients with BD from healthy controls. The discriminative efficacy of DKI-derived parameters and CBF values of the genu of the corpus callosum **(A)**, splenium of the corpus callosum **(B)**, temporal white matter **(C)**, and thalamus **(D)** were assessed for distinguishing the patients with BD from healthy controls.

Using ROC analysis, non-lesion areas of patients with BD were investigated to determine whether DKI/ASL-derived parameters could provide robust discrimination between patients with BD and healthy controls (Figure 4). Large area under the curves (AUCs) were identified in FA/FAk/Kr of the GCC (AUC: 0.957/0.908/0.752, *p* < 0.001, Figure 4A), CBF/FA/Dr/FAk of the SCC (AUC: 0.946/0.848/0.874/0.826, *p* < 0.0001, Figure 4B), MD/Da/Ka of the Tha (AUC: 0.942/0.933/0.7584, *p* < 0.0001, Figure 4D), as well as the combined FA/MD/Dr/CBF of the TWM (AUC: 0.986, *p* < 0.0001, Figure 4C), indicating high accuracy in distinguishing patients with BD from healthy controls. More detailed results are presented in Supplementary Figure 3.

## Discussion

This study investigated the diagnostic value of the combined 3D-pcASL and DKI in BD. Significant alterations were found with diffusion, kurtosis, and perfusion-related parameters in all WMHs, genu/splenium of the corpus callosum (GCC/SCC), and temporal WM (TWM) of patients with BD compared with healthy controls. Furthermore, kurtosis parameters, i.e., Ka and Kr, also showed significant changes in the right Cau, putamen, and thalamus within WM normal areas of patients with BD. In addition, using ROC analysis, high AUC values were identified for FA, MD, Dr, FAk, Kr, and CBF in the genu/splenium of the corpus callosum (GCC/SCC), thalamus, and temporal WM, indicating high accuracy in distinguishing patients with BD from healthy controls. Although the combined parameters did not show superior performance to each separate parameter on regions of GCC, SCC, and thalamus, we did find that the combined FA/MD/Dr/CBF in TWM showed the best diagnostic efficacy with the highest AUC (0.986). We consider this as an advantage of combining both 3D-ASL and DKI in the diagnosis of BD.

In this study, we manually selected ROIs in functional subregions of the brain and used traditional analysis of variance and *t*-test for statistical data analysis. The ROI selection scheme and analytic methods applied in this study were followed by some similar studies in the literature but focused on other degenerated diseases, e.g., PD (Wei et al., 2016).

Previous studies have proven that DKI is more sensitive than DTI in monitoring the alterations of brain microstructures closely linked with cognitive dysfunction in Parkinson’s disease (Wang et al., 2011) and Alzheimer’s disease (Gong et al., 2013). However, until now, the studies with quantitative DKI measurement for patients with BD are few. In this study, ensured by high measurement reliability of all DKI/ASL metrics, some of our results are consistent with the findings reported in previous DTI studies for patients with BD (Jung et al., 2014; Lee et al., 2018). FA was significantly declined, and diffusion parameters (i.e., MD, Da, and Dr) were significantly increased in WMHs and GCC in patients with BD compared with healthy controls, which indicates that fiber structures such as myelin sheath and glial cells in these areas were severely damaged, leading to damaged circuit connection area between the cortex. Unlike previous findings, kurtosis parameters of FAk and Kr in all WMHs, GCC, and SCC were significantly reduced in patients with BD. These results might indicate that (1) DKI is more sensitive in monitoring the potential damage of WM; (2) a complex microenvironment with highly restricted diffusion of water molecules was formed in the early stage of BD; and (3) serious damage occurred in both frontal cortex-subcortical connection and occipitotemporal subcortical connection of patients with BD.

All subjects showed higher Kr than Ka, which was consistent with the three-dimensional ellipsoidal water molecule motion theory. An interesting finding was that the decrease of Kr value was slightly larger than that of Ka among the three groups (i.e., WMHs, WM normal areas, and healthy controls). A plausible explanation for this may be that the rupture of the myelin membrane did not notably influence axial diffusion but significantly limited the diffusion of water molecules in radial non-normal distribution due to greatly increased radial water permeability (Cheung et al., 2009). MK is generally considered to lack sensitivity in detecting WM lesions. However, in our study, MK in some WM regions (centrum semiovale, frontal, and occipital WM around bilateral ventricle) showed significant differences among the three groups (i.e., WMHs of patients with BD, WM normal areas of patients with BD, and healthy controls). The reason for this may be that the damages to patients with BD in these areas were more serious, and the structures of these regions were relatively loose.

The statistical results showed that MK and Kr were significantly declined in BV-FWM normal areas of patients with BD compared with healthy controls. Similarly, Ka and Kr were also significantly declined in BV-OWM normal areas. In particular, Kr was more sensitive in distinguishing the WM lesions, WM normal areas, and the corresponding areas in healthy controls. In addition, Kr in BV-FWM of patients with BD was positively correlated with the MMSE score. But, the significant difference between the above regions was not found in diffusion parameters. These results indicate that the kurtosis parameters (i.e., MK, Ka, and Kr) can effectively distinguish the WM normal areas in BD from the WM normal areas in healthy controls. Furthermore, for patients with BD, Kr is useful in monitoring the BV-FWM damage and the progression of cognitive dysfunction.

3D pseudo-continuous arterial spin labeling is a fast spin-echo-based sequence with spiral sampling for high signal-to-noise ratio (SNR; Hernandez-Garcia et al., 2019) as well as three measurement repetitions (NEX = 3) were applied. We thus assume that a sufficient imaging SNR was obtained. 3D-pcASL is now a well-established MRI method for assessing cerebral perfusion in a quantitative manner (Jezzard et al., 2018). A previous study reported that 3D-pcASL can be considered an effective biomarker to evaluate functional changes of brain subregions for mild cognitive impairment patients (Dolui et al., 2017). In addition, ROI-based flow quantification derived from 3D pcASL has also been validated in the application of clinical diagnosis of WM diseases (e.g., LA; Bastos-Leite et al., 2008; Mandell et al., 2008; Skurdal et al., 2015; Zhong et al., 2017; Dolui et al., 2021), for clinical diagnosis and assessment in WM diseases. Based on these results, we assumed that ASL might hold potential in WM disease diagnosis and would like to investigate this in this study. However, the applied 3D-pcASL imaging with a single PLD of 1.5 s and labeling duration of 1.45 s might introduce limited SNR for CBF measurement in this study, especially for superior brain regions. This might partially explain why the CBF in temporal WM was higher than parietal WM. Moreover, due to not high in-plain image resolution applied in 3D-pcASL (e.g., 3.64 mm × 3.64 mm), the partial volume effect, especially for small ROIs close to ventricles, might not be neglected. We thus consider this as a methodological limitation of this study.

In this study, CBF was significantly decreased in all ROIs, including WM areas, basal ganglia (right caudate nucleus and putamen), thalamus, and right hippocampus, of patients with BD. Additionally, the area with reduced CBF was larger than that with WM damage. The explanation for this may be that deep perforating arterial wall hardening, glial cell hyperplasia, and vascular endothelial cell dysfunction first occurred in the early stage of BD. The secondary inflammation alterations may also occur in these arterial walls, leading to the narrowing of the lumen and reduced CBF in large area (Rigsby et al., 2007). Furthermore, for patients with BD, the CBF values in WM around the bilateral ventricle were positively correlated with FA and Kr values. As the decreased FA and Kr often reflect demyelination and axonal loss of WM, the positive correlation indicates that low microcirculation might play an important role on the demyelination of deep WM.

In this study, we found that some normal-performing regions on FLAIR showed significant changes with part of DKI and ASL parameters. In addition, previous studies (Chai et al., 2018; Thaler et al., 2021) have demonstrated that the parameters of DKI and ASL can be more sensitive and comprehensive than FLAIR in monitoring WM lesions. With these findings, the combination of DKI and ASL may be able to detect the underlying lesions of WM normal areas shown on FLAIR. However, we consider that it could improve the current work with additional analysis of FLAIR on diagnosing patients with BD. We thus consider this in our follow-up study.

Yin et al. (2014) concluded that patients with BD exhibit atrophy in the hippocampus and medial temporal cortices, and the atrophy in medial temporal cortices and deep gray matter may be the unique pathological basis of cognitive impairment. In this study, for patients with BD, CBF of parietal/temporal WM, and FA/Kr of frontal WM around bilateral ventricle were positively correlated with MMSE score. Strong negative correlations were found in the thalamus between CBF and MD and between Da and MMSE scores. These results indicated that the hypoperfusion of parietal/temporal WM, the damage of frontal WM around the bilateral ventricle, as well as the hypoperfusion and damage of the thalamus nerve bundle in the axial position might play important roles in the cognitive dysfunction of patients with BD. In addition, for patients with BD, although FA, MK, Ka, and Kr values of the right hippocampus were reduced, no significant difference was found with healthy controls. The significantly reduced CBF was found in the right hippocampus of patients with BD, but no significant correlation was found with the MMSE score. This observation indicates that patients with BD may suffer less damage to mood and memory function.

To seek robust diagnostic capabilities, we used ROC analysis on WM normal areas of patients with BD. We found that FA/FAk/Kr in GCC, CBF/FA/Dr/FAk in SCC, MD/Da/Ka in thalamus, and the combined FA/MD/Dr/CBF values in temporal WM were all highly accurate in distinguishing patients with BD from healthy controls. These highly accurate diagnoses indicate that although diffusion-related parameters may be sensitive indicators in the diagnosis of patients with BD, kurtosis-related parameters and CBF value can also provide added clinical value in the diagnosis of BD.

This study has limitations. First, the number of participants was relatively small and the number of men and women did not perfectly match. A large sample size is required for further clinical validation. Second, due to the low image resolution applied in DKI and ASL images, the partial volume effect could not be fully eliminated during ROI selections in each functional subregion of the brain, particularly for the smaller ROIs close to ventricles. Novel diffusion and ASL techniques, such as multi-shot-based diffusion imaging (Chen et al., 2013) for allowing high-image resolution without image distortion, voxel-based morphometry (VBM; Ashburner and Friston, 2000) for quantitatively calculating changes in local gray and WM density and volume, and ASL with multiple-PLDs (Choi et al., 2018; Cohen et al., 2020) for obtaining additional arterial transit time (ATT) map to present the blood flow velocity (low signal in superior brain regions might be sufficiently compensated), are thus needed in our follow-up study to address this issue. In addition, the additional analysis of FLAIR on diagnosing patients with BD, which can improve the current work, is thus also needed in our follow-up study.

## Conclusion

This study applied both DKI and ASL techniques in the diagnosis of patients with BD. The correspondingly derived diffusion, kurtosis, and perfusion parameters showed high sensitivities in detecting the alterations of microstructures in WMHs and WM normal areas of patients with BD. In addition, DKI and ASL images are beneficial to detect the progression of cognitive dysfunction in patients with BD. Finally, confirmed by high AUC, in addition to diffusion parameters of FA, MD, and Dr, kurtosis-related parameters (FAk, MK, and Kr) also provided robust performance in the diagnosis of patients with BD. Therefore, the combination of both DKI and 3D-ASL imaging techniques could be considered an effective tool in the clinical diagnosis of patients with BD.

## Data Availability Statement

The original contributions presented in this study are included in the article/Supplementary Material, further inquiries can be directed to the corresponding author.

## Ethics Statement

The studies involving human participants were reviewed and approved by the Medical Ethics Committee of The First Affiliated Hospital of Shandong First Medical University. The patients/participants provided their written informed consent to participate in this study.

## Author Contributions

HS was in charge of the experimental design and provided important suggestions in this study. XH was in charge of collecting patients, operating magnetic resonance imaging, statistical analysis, chart production, and article writing. WD was in charge of providing the overall idea, guidance, language polish, and revision opinions. All authors contributed to the article and approved the submitted version.

## Conflict of Interest

WD was employed by the company GE Healthcare, Beijing. The remaining authors declare that the research was conducted in the absence of any commercial or financial relationships that could be construed as a potential conflict of interest.

## Publisher’s Note

All claims expressed in this article are solely those of the authors and do not necessarily represent those of their affiliated organizations, or those of the publisher, the editors and the reviewers. Any product that may be evaluated in this article, or claim that may be made by its manufacturer, is not guaranteed or endorsed by the publisher.
