# Supplementary material for: The Diagnostic Value of the Combined 3D Pseudo-Continuous Arterial Spin Labeling and Diffusion Kurtosis Imaging in Patients With Binswanger’s Disease
Source: Front Neurosci. 2022 Jun 30;16:853422. doi: 10.3389/fnins.2022.853422 (PMC9280636; doi:10.3389/fnins.2022.853422)
Supplement: Supplementary file 2 [file Data_Sheet_2.docx]

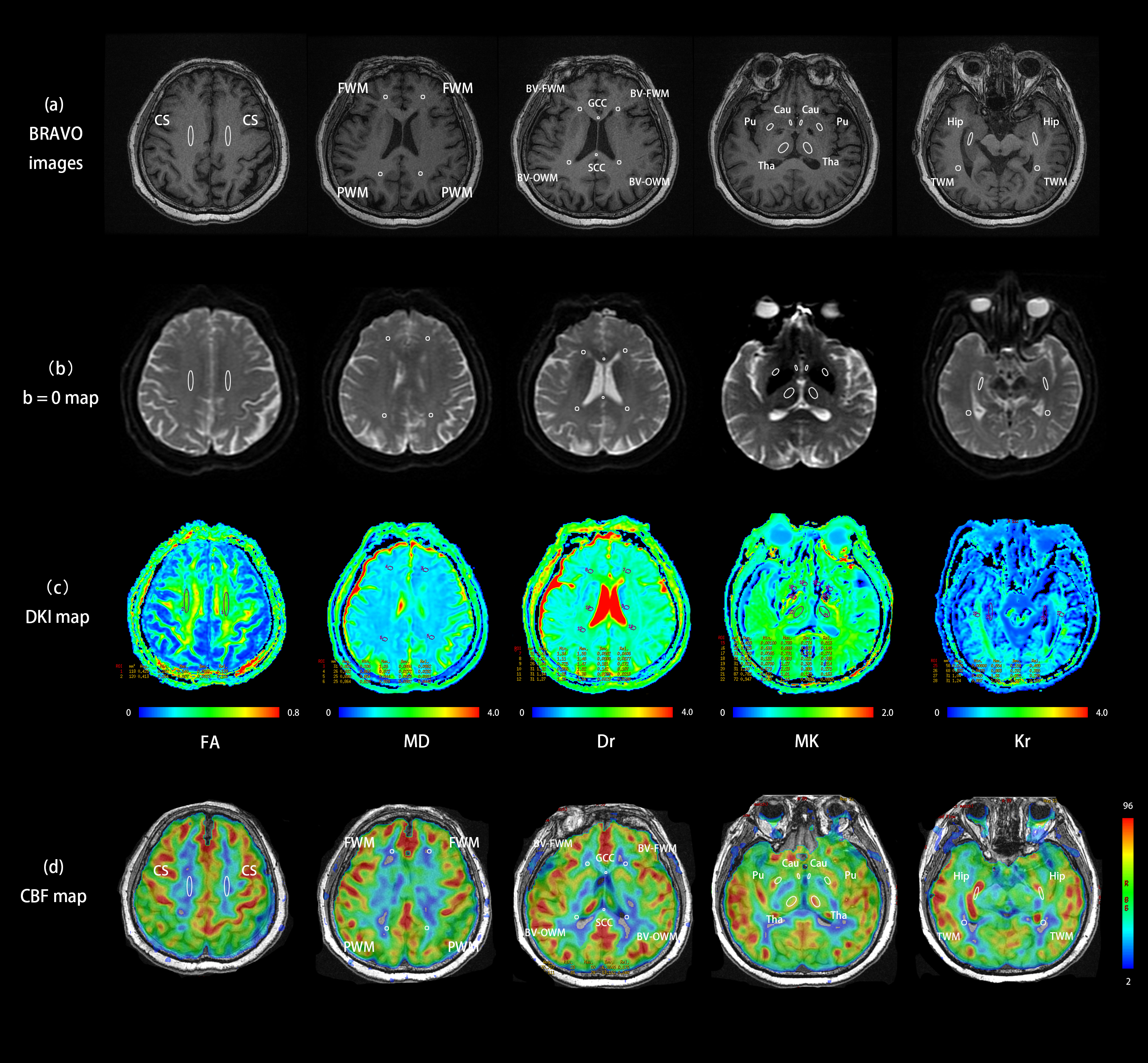


Figure ^sup^ 1. Representative MR images at five consecutive slices of a healthy control acquired using BRAVO image (a), DKI b = 0 map (b), DKI parametric maps (c) and CBF map (d).

ROI settings. CS, centrum semicovale; GCC/SCC, genu/splenium of the corpus callosum; FWM/PWM/TWM, frontal/parietal/temporal white matter; BV-FWM/BV-OWM, frontal/occipital white matter around lateral ventricle; Cau, caudate nucleus; Pu. Putamen; Tha, thalamus; Hip, hippocampus. Each ROI was also denoted with BRAVO images, b=0 maps, and DKI/ASL-derived parameters maps.


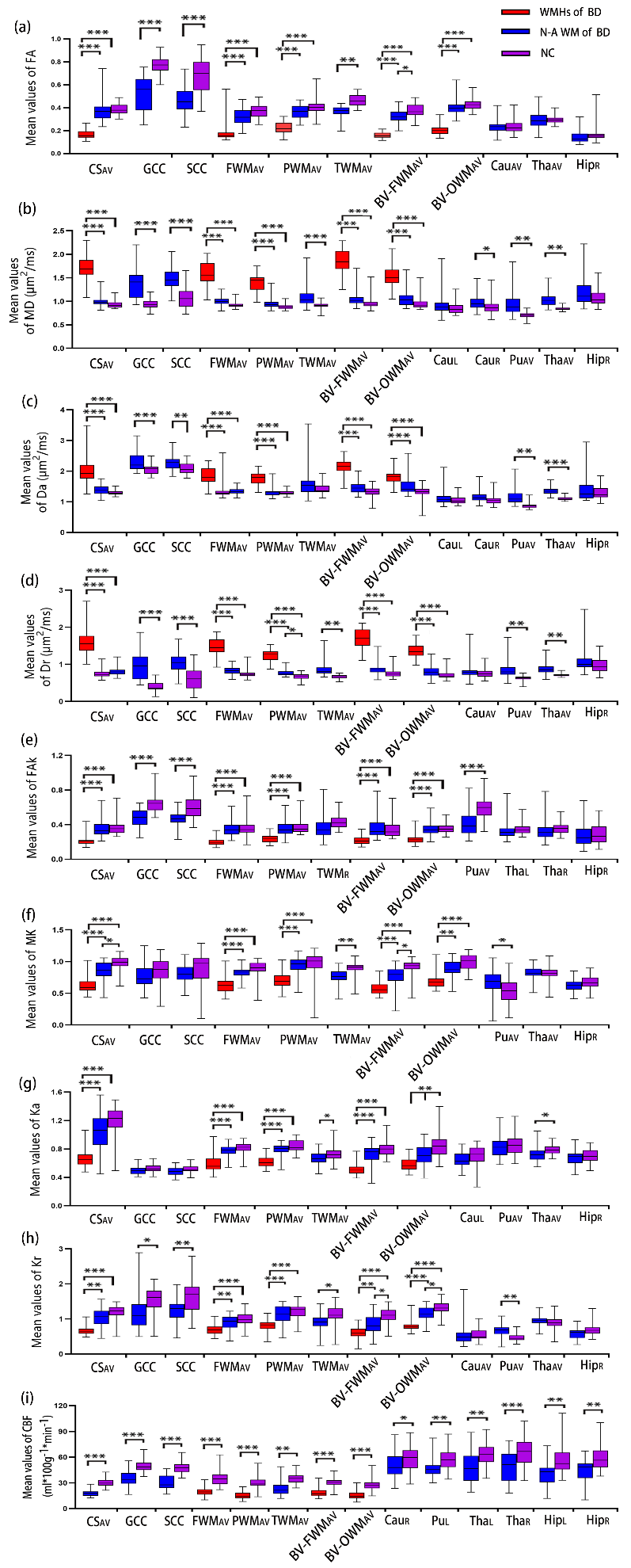


Figure ^sup^ 2. Box and whiskers graphs of DKI measurements (FA, MD, Da, Dr, FAk, MK, Ka, and Kr) among the three groups (WMHs of BD, WM normal-areas of BD, and NC) and ASL measurement (CBF) between BD and NC groups over different sub-regions of the brain. The solid line inside the box represents the median value, whereas the edges represent the 25th and 75th percentiles. Straight line (bar) on each box indicates the range of data distribution.

Note: BD, Binswanger’s disease; WMHs of BD, white matter hyperintensities of BD patients; N-A WM of BD, white matter normal-areas of BD patients; NC, healthy control; L, the left hemispheric side; R, the right-hemispheric side; AV, average of bilateral ROI measurements; CS, centrum semicovale; GCC/SCC, genu/splenium of the corpus callosum; FWM/PWM/TWM, frontal/parietal/temporal white matter; BV-FWM/BV-OWM, bilateral ventricle around frontal and occipital white matter ; Cau, caudate nucleus; Pu, putamen; Tha, thalamus; Hip, hippocampus. Significant difference: *, *p* < 0.005; **, *p* <0.001; ***, *p* < 0.0001.


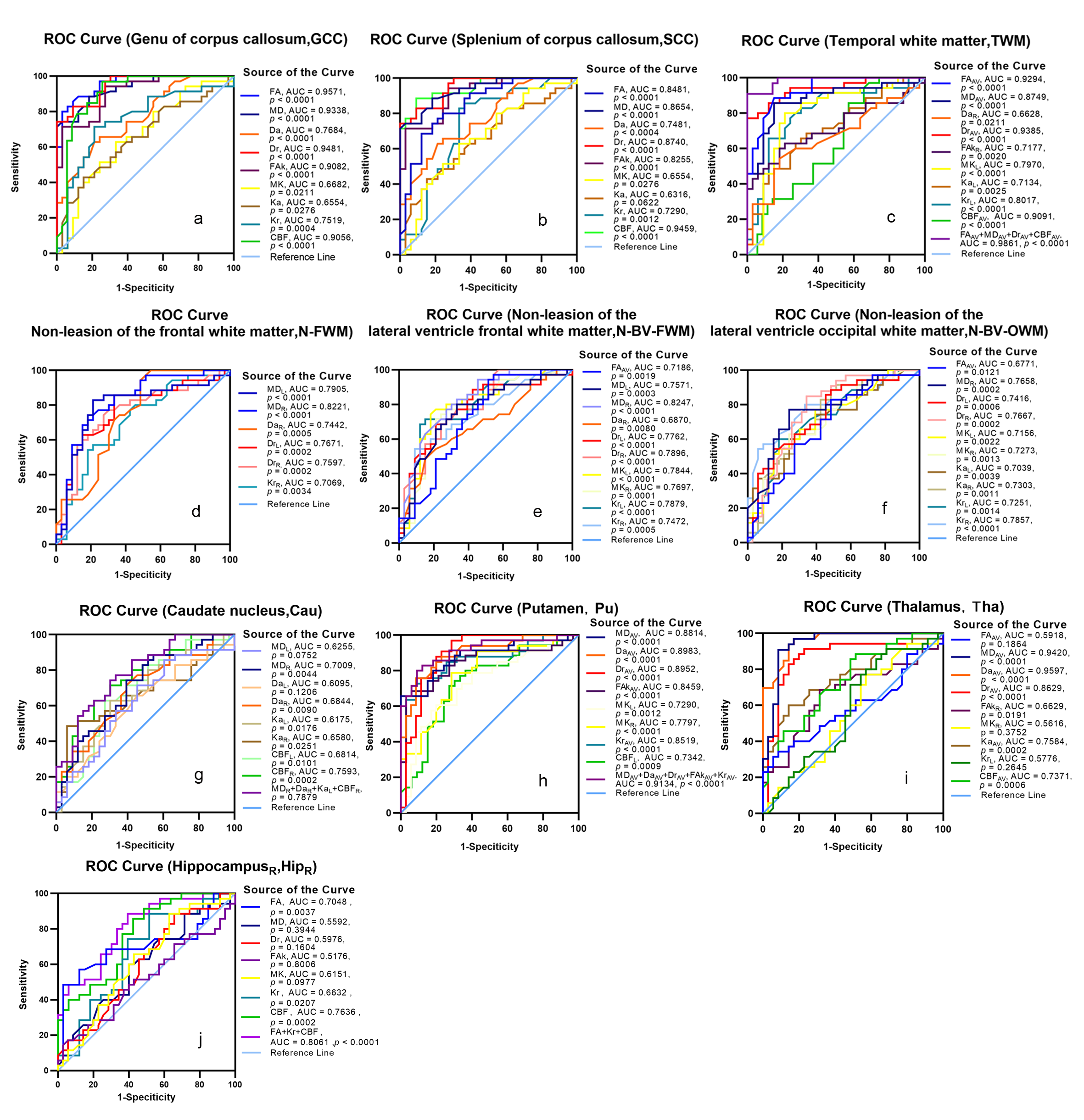


Figure ^sup^ 3. The ROC analysis to assess the efficacy of DKI/ASL-derived parameters for discriminating BD patients from healthy controls.

The diagnostic efficacies of DKI-derived parameters and CBF values of the genu of the corpus callosum (a), splenium of the corpus callosum (b), temporal white matter (c), frontal white matter (WM) normal areas (d), bilateral ventricle around frontal WM normal areas (e), bilateral ventricle around occipital WM normal areas (f), caudate nucleus (g), putamen (h), thalamus (i), and hippocampus (j) were assessed for distinguishing BD patients from healthy controls.
